# Wegener granulomatosis: type of presentation and initial treatment in a series of 23 cases

**DOI:** 10.1186/1479-5876-10-S3-P50

**Published:** 2012-11-28

**Authors:** Rosalia Martínez-Pérez, Mario Leon, Julia Uceda, S Rodriguez Montero, A Muñoz, ML Velloso, JL Marenco

**Affiliations:** 1 Rheumatology Service, Valme University Hospital, Sevilla, Spain

## Introduction

Granulomatosis with poliangeitis (Wegener) is a necrotizing granulomatous vasculitis characterized by damage in the respiratory tract, kidney, skin, nervous system ... The onset of the disease is usually indolent, with nonspecific symptoms. The most frequent initial clinical presentation is the upper respiratory tract involvement (90%). The presence of pulmonary infiltrates is 70% and bilateral and cavitary nodules in about 60%.

## Materials and methods

A retrospective study in a cohort of 23 patients diagnosed with Granulomatosis with poliangeitis (Wegener). We analyzed clinical variables that led to the admission and diagnosis of disease.

## Results

In our series the average age was of 48.4 ± 20.3 years, comprising 15 men and 8 women. The average age at diagnosis was 43.4 ± 18.6 years. All patients were positive c-ANCA and PR3 specific, and required hospitalization at the onset, presenting as reason for admission the following symptoms. See Table 1.

**Table 1:** 

	Fever	Arthritis	Oral	Cutaneous	Pulmonary	Renal	Neurological
**Patients** (%/absolute Number)	82.6% /19	47.8% /11	65.2% /15	47.8% /11	56.5% /13	34.8% /8	30.4% /7

Eight patients debuted with early renal impairment in urinary sediment (hematuria and proteinuria in nephrotic range) without impact on renal function, which resolved after administration of intravenous steroids. In 34.8% (8 patients) was observed pulmonary involvement by the presence of multiple cavitary pulmonary nodules and bilateral. Initial treatment was in 21.7% only prednisone, prednisone in 65.2% with cyclophosphamide, in 4.3% was added azathioprine and in 8.7% required Rituximab for severe pulmonary involvement. The overall trend was positive in 100% of cases after establishing high-dose glucocorticoid therapy, except in the case of a patient who presented with intestinal vasculitis with diffuse enteritis and multiple intestinal perforations, requiring emergency surgery.

## Conclusions

With this study, we show that the clinic that motivates the initial admission and diagnosis of these patients is acute, nonspecific, with a spectrum of varying severity, with the most frequent involvement of cutaneous-articular and upper respiratory tract, such as recorded in the scientific literature. Sometimes the clinical presentation may evolve fastly into forms of serious illness, which requires early diagnosis, allowing early start of appropriate immunosuppressive treatment.

